# Acceptability of a feasibility randomized clinical trial of a microenterprise intervention to reduce sexual risk behaviors and increase employment and HIV preventive practices (EMERGE) in young adults: a mixed methods assessment

**DOI:** 10.1186/s12889-020-09904-x

**Published:** 2020-12-02

**Authors:** Larissa Jennings Mayo-Wilson, Jessica Coleman, Fatmata Timbo, Carl Latkin, Elizabeth R. Torres Brown, Anthony I. Butler, Donaldson F. Conserve, Nancy E. Glass

**Affiliations:** 1 Department of Applied Health Science, Indiana University School of Public Health, 1025 E. 7th Street, Bloomington, IN 47405 USA; 2 Department of International Health, Johns Hopkins University School of Public Health, 615 N. Wolfe Street, Baltimore, MD USA; 3 HEBCAC Youth Opportunity (YO!) Program, Johns Hopkins University School of Medicine, 1212 N. Wolfe Street, Baltimore, MD USA; 4 AIRS, Inc., Empire Homes of Maryland, Inc., City Steps, 1800 N Charles Street, 7th Floor, Baltimore, MD USA; 5 Department of Health Promotion, Education, and Behavior, Arnold School of Public Health, University of South Carolina, 915 Green Street, Columbia, SC 29208 USA; 6 Johns Hopkins University School of Nursing, 525 N. Wolfe Street, Baltimore, MD USA

**Keywords:** HIV, Sexual risk behaviors, Microenterprise, Text messages, Young adults, Baltimore, Economic, Acceptability, Clinical trial, Qualitative, African-American

## Abstract

**Background:**

Acceptability is a critical requisite in establishing feasibility when planning a larger effectiveness trial. This study assessed the acceptability of conducting a feasibility randomized clinical trial of a 20-week microenterprise intervention for economically-vulnerable African-American young adults, aged 18 to 24, in Baltimore, Maryland. *E*ngaging *M*icroenterpris*E* for *R*esource *G*eneration and Health *E*mpowerment (EMERGE) aimed to reduce sexual risk behaviors and increase employment and uptake of HIV preventive behaviors.

**Methods:**

Thirty-eight participants were randomized to experimental (*n* = 19) or comparison group (n = 19). The experimental group received text messages on job openings plus educational sessions, mentoring, a start-up grant, and business and HIV prevention text messages. The comparison group received text messages on job openings only. Qualitative and quantitative post-intervention, in-person interviews were used in addition to process documentation of study methods.

**Results:**

Our results found that the study design and interventions showed promise for being acceptable to economically-vulnerable African-American young adults. The largely positive endorsement suggested that factors contributing to acceptability included perceived economic potential, sexual health education, convenience, incentives, and encouraging, personalized feedback to participants. Barriers to acceptability for some participants included low cell phone connectivity, perceived payment delays, small cohort size, and disappointment with one’s randomization assignment to comparison group. Use of peer referral, network, or wait-list designs, in addition to online options may enhance acceptability in a future definitive trial. Expanding administrative and mentoring support may improve overall experience.

**Conclusion:**

Microenterprise interventions are acceptable ways of providing young adults with important financial and sexual health content to address HIV risks associated with economic vulnerability.

**Trial registration:**

ClinicalTrials.gov. NCT03766165. Registered 04 December 2018.

## Background

Microenterprise interventions, often referred to as interventions to launch very small-scale businesses, are a relatively new potential strategy for prevention of HIV in vulnerable populations given their focus on economic drivers of sexual risk-taking [1]. Research has shown that economic vulnerability, such as homelessness and unemployment, contributes to HIV risk due to costs in accessing HIV preventive services [2–5], exposure to sexual violence [6–8], reliance on high-risk sex work for money, food, or housing [9–12], misinformation regarding HIV in underserved communities [13, 14], and diminished motivations to avoid HIV due to negative psychological consequences of financial distress [15, 16]. HIV prevalence is 2.1 times higher in persons with income equal to or lower than the U.S. poverty threshold, and 2.6 times higher in persons who lack employment [17, 18]. However, little is known about the feasibility and effectiveness of microenterprise interventions in U.S. concentrated areas of poverty.

In addition, HIV-focused microenterprise interventions have been understudied among U.S. racial minorities, despite these populations being disproportionately affected by HIV [19]. African-Americans, who make-up a substantial proportion of the urban homeless and unemployed [20], have a rate of new HIV infections that is 8.3 times higher than that of non-Hispanic whites [20]. In fact, health disparities by race in the U.S., including HIV, are well-documented [19–24]. These disparities are, in part, driven by the overlapping factors of economic deprivation and racism [20, 22, 25–27]. In addition to interpersonal racism with prejudices in the hiring of African-Americans, structural racism in the form of segregating and discriminating policies has led to vast economic inequalities with a large proportion of African-Americans living in highly economically-deprived urban areas with high rates of unemployment and adverse health outcomes compared to their U.S. white counterparts [20, 22, 26, 27]. To reduce HIV and other health disparities and to reduce the long-term impacts of structural racism, it is critical to address economic status in African-American communities, particularly income and asset generation among African-American young adults. Yet, with a few exceptions, most microenterprise interventions have been conducted outside of the U.S. in low-income countries [28–36]. The absence of evidence has limited the development and the implementation of programs to address economic disparities and its impact on HIV prevalence in this population.

This article describes an acceptability study undertaken as part of a larger assessment of the feasibility of conducting a randomized clinical trial of a 20-week microenterprise intervention [37]. *E*ngaging *Mi*croenterpris*E* for *R*esource *G*eneration and Health *E*mpowerment (EMERGE) aimed to reduce sexual risk behaviors and increase employment and uptake of HIV preventive behaviors in economically-vulnerable African-American young adults (clinicaltrials.gov NCT03766165). The overall aim of the feasibility trial was to assess five domains: participant recruitment, randomization, participation, retention, and acceptability. Progression criteria based on findings across all domains were developed to determine whether and how to proceed to a full-scale trial, such as the ability to reach the target sample, achieving ≥70% study participation, minimizing loss-to-follow-up, and having sufficient acceptability [37]. A previously published main outcomes manuscript reports on quantitative progression criteria from the researcher’s perspective as it related to recruitment, randomization, retention, and participation, in addition to changes in sexual and economic outcomes [38]. As part of the feasibility trial, this manuscript focuses on the final progression criteria of acceptability by obtaining qualitative aspects of acceptability from participant and stakeholder perspectives as it related to the study’s interventions (e.g., comparison and experimental) and the study’s assessment method (e.g., weekly text message surveys). Specifically, we obtained qualitative feedback and quantitative ratings regarding what individuals liked and disliked regarding the study, perceived benefits and harms, ease of use, referral to others, and recommendations for improvements. Acceptability is a critical requisite in establishing feasibility when planning a larger effectiveness trial [39, 40]. To our knowledge, few mixed methods studies have reported on the experiences of economically-vulnerable U.S. young adults who participated in an HIV prevention microenterprise trial.

## Methods

### Design

We examined acceptability of a randomized clinical trial with a two-group parallel design and 1:1 allocation ratio to experimental or comparison group. The trial is registered at ClinicalTrials.gov (NCT03766165). Mixed methods post-intervention interviews were conducted to examine acceptability among study participants, mentors, and managers using quantitative ratings and qualitative open-ended questions. We used a triangulation convergence design in which quantitative and qualitative data were collected concurrently, analyzed with equal weight, and merged during interpretation [41, 42]. Mixed methods were used to obtain different but complementary acceptability data that enumerated participants’ points of view while documenting open feedback [41, 42].

### Setting

The location off the study was Baltimore, Maryland (MD). African-Americans make up the majority (82%) of adult and adolescent HIV diagnoses in Baltimore, MD [43], and Baltimore young adult residents, 20–29 years old, account for the largest percentage of HIV diagnoses (29%) in comparison to other age categories [43]. The study was conducted in collaboration with two community-based organizations (CBOs), AIRS and YO!Baltimore, offering supportive housing to young adults who have experienced residential instability.

### Participants

**Fig. 1:**
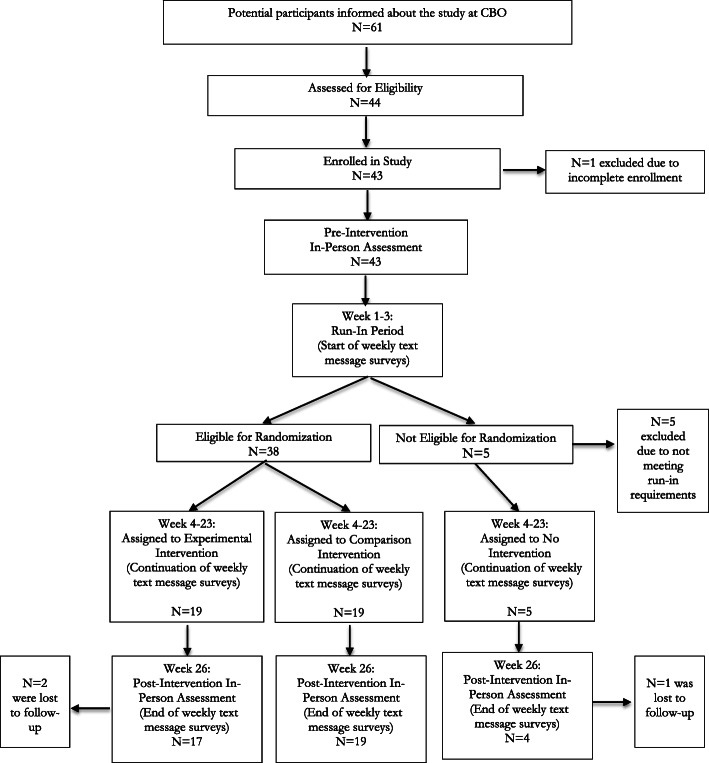
Participant Flow Diagram

We have published the study’s participant recruitment process in a publicly-available protocol manuscript [37]. A standard CONSORT diagram is shown in Fig. 1. In summary, a screening tool was used to assess study eligibility in-person at the time of enrollment. Participants were eligible if they: were living in Baltimore, were 18 to 24 years old, were African-American, had experienced one or more episodes of homelessness in the last 12 months (e.g., defined as reporting any episode in which a person lacked a regular or adequate nighttime residence, such as living in a hotel/motel, vehicle, shelter, or friend’s home and living primarily on their own, apart from a parent or guardian), were unemployed or underemployed (e.g.,< 10 h per week), were not enrolled in school, owned a cell phone with text messaging, and reported one or more episodes of unprotected sex in the last 12 months.

We recruited participants from the study’s two participating CBOs. Potential participants were notified about the study team’s scheduled visit days by staff at the CBO using recruitment flyers, emails, and/or word-of-mouth. Next, the PI and/or a trained research assistant introduced the study’s goals to interested young adults on those visit days and privately administered written informed consent. At this time, we also registered the cell phone of each participant to the study’s text messaging program (TextIt.in) prior to carrying out the pre-intervention assessment.

### Interventions

**Fig. 2:**
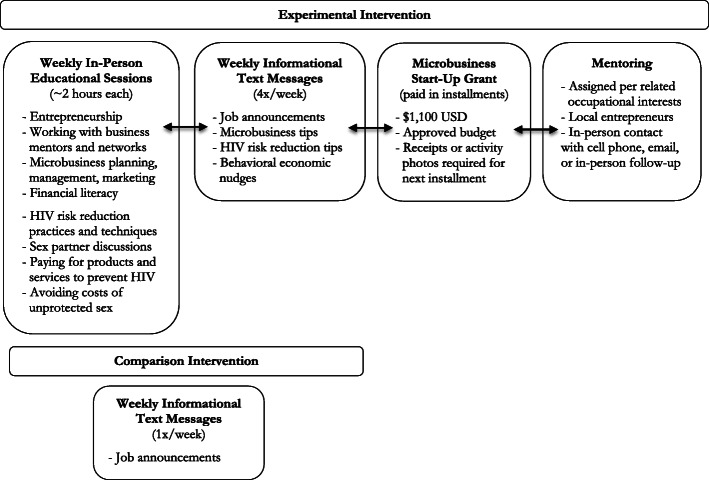
Components of the 20-Week EMERGE Experimental and Comparison Interventions

Figure 2 depicts core intervention activities. Specific information regarding the interventions and text message assessments are published in the aforementioned protocol manuscript [37]. In summary, the experimental intervention participants were provided: (1) a weekly text message each on local job announcements every Monday; (2) a weekly two-hour educational classroom-based session relating to HIV prevention and microbusiness start-up on Wednesdays; (3) an assigned mentor according to each participant’s microbusiness interests; 4) a microgrant of $1100.00 USD; and (5) three weekly text messages relating to microenterprise and HIV prevention. The EMERGE project also included frequent rewards such as certificates, cupcakes, words of affirmation, and recognition to participants during weekly educational sessions for achieving milestones such as acquiring grant payment, obtaining a first client, earning first profits, or acquiring an internship. To reduce contamination, participants assigned to the experimental intervention were asked to refrain from talking about the intervention to peers assigned to the comparison intervention. Comparison intervention participants were provided only the identical weekly job announcement text messages.

All participants were asked to respond to a weekly text message survey over the course of the study period on Fridays. The text message survey included 16 questions (identical each week) that assessed sexual behaviors, use of HIV preventive practices, and employment outcomes. Participants received a $3 cash payment every Tuesday for responding to the previous week’s text message survey. This was provided each week by leaving sealed envelopes with cash and labeled with participant’s phone numbers with the CBO manager. Responders then received a text message reminder to pick-up their payment at the CBO desk office.

### Timeline

Recruitment was conducted from December 2018 to February 2019. Participants in both groups received the assigned interventions and weekly text message surveys concurrently for 20 weeks from February to July 2019. Participants additionally completed one in-person, post-intervention interview from July to August 2019. Process documentation in the form of checklists and note-taking on lessons learned by the study team was conducted throughout the study period from December 2018 to August 2019.

### Progression criteria to definitive trial

This feasibility trial relied on several progression criteria relating to recruitment, randomization, retention, and participation, in addition to acceptability of the study’s interventions and assessment method. As published in the main outcomes manuscript, the study reached its recruitment target (100%), randomized all participants who completed the run-in requirements [37] (88% completed), and maintained high retention (93%) [38]. We also previously published participation and behavioral outcomes [38]. In sum, 71% of experimental participants attended educational sessions in the first half of the study (weeks 1 to 10), although attendance declined to 35% in the latter half (weeks 11 to 20) [38]. Approximately 58% of participants engaged in mentoring and microbusiness grant spending, and 82% of participants responded to the text message survey in the first half of the study (weeks 1 to 10), although response rates declined to 45% in the latter half (weeks 11 to 20) [38]. In determining whether and how to proceed to a fully powered effectiveness trial, this manuscript reports on progression implications relating to acceptability of the study in the context of design, interventions, and outcome assessments.

### Data collection

Acceptability data were collected using two methods: (1) individual interviews conducted at the end of the interventions with participants, mentors, and CBO managers; and (2) a process documentation file of web-based notes and lessons learned that were added to over the course of the study by the implementation team (LJMW, JC, FT) from interactions with participants, mentors, CBO managers, and co-investigators. Post-intervention interviews aimed to gather feedback on study acceptability from multiple points of view. Use of individual interviews rather than focus group discussions was also intended to encourage active contributions by all participants with minimal distraction from others. Process documentation aimed to record insights observed while the study was underway and potential modifications needed in a future effectiveness trial.

Post-intervention interviews with participants were conducted using a semi-structured interview guide developed by the study team that included qualitative open-ended questions (e.g., no pre-coded responses) and quantitative close-ended questions with pre-coded categories [Supplementary File 1]. The qualitative questions were asked at the beginning of the interview to document factors not previously considered or known. These questions asked participants to describe what they liked and disliked about being a part of the EMERGE project, including various aspects of the interventions and the study’s assessment activities. We also asked participants to describe what changes, if any, they would like to see implemented if they were selected again as EMERGE participants, including recommendations along the continuum of recruitment to implementation to follow-up. The qualitative open-ended questions were intentionally broad to make the topics as accessible as possible. Data were recorded using written field notes only. As the aim of the inquiry was not a purely qualitative one, use of field notes facilitated a rapid and cost-efficient analysis [44–46]. Interviewers made written notes of short-hand quotations as the interview progressed and then added to these notes and expanded quotations once the interview was completed.

Participants were also encouraged to respond freely to a set of quantitative closed-ended questions that aimed to tally participant views on acceptability of the interventions and study assessments. We asked six questions about acceptability, in terms of likability, perceived helpfulness, ease of use, and referral of the interventions to others. These questions were: How much did you like the intervention?; How much help to you was the intervention in improving your ability to earn income?; How much help to you was the intervention in improving your ability to prevent HIV?; How likely are you to recommend the intervention to a friend?; How much did you like the text message survey?; and How easy was it to respond each week to the text message survey?. A three-point Likert scale (e.g., very helpful, somewhat helpful, not helpful) was used for all of the acceptability questions. A final set of quantitative, closed-ended questions asked participants about their receipt and use of weekly job announcement text messages and preferences for more or fewer text messages. Participants were provided snacks and $20 in cash immediately after the interview. Demographic data relating to age, gender, education, employment status, parental status, prior night’s residence, and income insecurity (e.g., having enough money to buy food, housing, and/or transportation in the last 30 days) were collected at baseline for all enrolled participants.

Post-intervention interviews with CBO managers and mentors used an open-ended interview guide that similarly focused on opinions about the strengths and limitations of the interventions and recommendations, if any, for improvements in a future effectiveness trial. Field notes along with transcriptions from audio-recordings were used given the more in-depth discussion. All interviews were conducted in English and lasted an average of 20 to 30 min. CBO manager interviews were carried out in person at the CBO site, recorded, and transcribed. Mentor interviews were carried out over the phone to account for varying business schedules with responses documented using written field notes. No payments for interviews were provided to mentors or CBO managers.

### Sample size

The target sample size for assessing acceptability was all randomized participants (*n* = 38), CBO managers (n = 3), and individual microbusiness mentors (*n* = 8) who participated in the study. A universal sample of randomized participants was chosen as it was feasible given the small size and was most suitable in maximizing variability. We anticipated that one interview with each participant or stakeholder would be sufficient to reach saturation, in which no new information was identified.

### Analysis

Descriptive statistics using frequencies and proportions were used for all quantitative acceptability questions and compared across study groups. For qualitative acceptability questions, a content analysis was used to identify patterns in participants’ statements and produce qualitative description [47, 48]. The goal of the qualitative content analysis was to describe the acceptability findings rather than make any explanatory links between categories [47, 48]. First, we repeatedly read and reviewed all participants’ expanded field notes and short-hand quotations, which made up the data from which analyses were performed. We entered each participant’s responses into a study-generated Excel database across three topics: reported likes, dislikes, and recommendations. Next, we developed a codebook consisting of pre-determined study activities relevant to each study group (e.g., educational sessions, mentoring, grants/incentives, informational text messages, text message surveys, and miscellaneous) to code participant responses. We then grouped responses within each code and topic to describe the overall opinion. Exemplary quotations were extracted to support findings. A similar process was used for interviews with the mentors and CBO managers. To provide a sense of the prevalence of some opinions relative to others, we also tallied the number of participants who mentioned each opinion. As a final step, lessons learned from the process documentation were synthesized. The synthesis process involved an initial reading and re-reading of study materials, notes, and CBO correspondences to identify key lessons learned. We also analyzed lessons learned from weekly discussions with the implementation team. These discussions included review of study progress, data availability, implementation facilitators, identification of delays or barriers and efforts to address them, as well as potential adaptions needed in the current feasibility trial or in a future effectiveness trial.

## Results

### Participant characteristics

Table 1 reports demographic characteristics of all randomized participants. The mean age was 21.1 years. Thirty-four percent (34%) were male. Most (76%) had a high school diploma or equivalent as their highest level of education. Unemployment and income insecurity were high (84 and 82%, respectively) (Table 1). Housing status varied with 18% of randomized participants having spent the previous night in an emergency shelter compared to 3% with a stranger; 40% in transitional housing; and 34% at the home of a friend, relative, or intimate partner. Five percent (5%) had their own apartment. Thirteen percent (13%) were biological parents. Acceptability data were obtained for 95% (*n* = 36) of randomized participants. This represents 89% (*n* = 17) of experimental participants and 100% (*n* = 19) of comparison participants. Acceptability data were obtained for 64% (*n* = 7) of stakeholders: 100% (n = 3) of CBO managers and 50% (*n* = 4) of individual microbusiness mentors.

**Table 1:** Baseline demographic characteristics of randomized study participants (*N* = 38) in the EMERGE feasibility randomized clinical trial by group and total

Characteristic	Study Group		Total
	Experimental	Comparison	
Number of enrolled participants	–	–	43
Number of enrolled participants who were not randomized	–	–	5
Number of enrolled participants who were randomized	19	19	38
Mean age in years	21.3	20.9	21.1
Age range in years (min, max)	18, 24	18, 24	18, 24
Male	32%	37%	34%
Highest level of education			
Grades 8 to 11	32%	16%	24%
High school diploma	63%	84%	74%
2-Year College	5%	0	2%
4-Year College	0	0	0
Unemployed	84%	84%	84%
Income insecurity in last 30 days	90%	74%	82%
Previous night’s residence			
Emergency shelter (CBO)	32%	5%	18%
Transitional housing (CBO)	37%	42%	40%
With friend, relative, partner	26%	42%	34%
With stranger	0	5%	3%
Street/public space	0	0	0
Private apartment	5%	5%	5%
Currently a parent	16%	11%	13%
Completed a post-intervention assessment of acceptability	17 (89%)	19 (100%)	36 (95%)

### Acceptability of job announcement text messages

Table 2 summarizes quantitative acceptability ratings of several intervention activities, including the weekly job announcement text messages. The majority (84%) of comparison participants reported that they liked the job announcement “a lot” compared to 11% who reported “somewhat liking” the job announcement and 5% who stated that they “did not like” the job announcements (Table 2). In assessing the perceived effectiveness of the comparison intervention, 47% reported that the job announcements were “very helpful” in improving their income-earning ability compared to 37% who reported that the job announcements were “somewhat helpful” and 16% who reported that they were “not helpful.” About half (47%) of comparison participants stated that they applied to one or more texted job announcements, and 22% of those who applied reported receiving one or more jobs. Fifty-three percent (53%) of comparison participants stated they would prefer to receive equal number of job announcement text messages in a future intervention compared to 21% who would prefer to receive more job announcement text messages and 26% who would prefer to receive fewer. Seventy-one percent (71%) of experimental participants reported wanting equal number of text messages in a future intervention compared to 12% who wanted more text messages and 18% who wanted fewer text messages. Qualitative comments regarding the weekly job announcement text messages further iterated preferences for more job postings among participants and adding a study facilitator to assist participants in applying to texted jobs of interest.

“What I liked most about EMERGE were the weekly job announcements…all the updates. If I could change one thing it would be to have more job announcements…” – Woman, Comparison

“With the job announcements, you could also bring in an employment person to help the residents. It would be like having this person assigned only to help with the employment needs.” – CBO Manager

**Table 2:** Responses to quantitative acceptability questions on intervention helpfulness among n = 36 participants in the EMERGE feasibility randomized clinical trial by group and total

Acceptability question	Study Group		Total
	Experimental	Comparison	
Number of enrolled participants who were randomized	19	19	38
Number of randomized participants who completed a post-intervention assessment of acceptability	17	19	36
Extent of liking the intervention^a^			
Liked a lot	100%	84%	92%
Somewhat liked	0	11%	6%
Did not like	0	5%	3%
Helpfulness in improving income-earning ability			
Very helpful	53%	47%	50%
Somewhat helpful	47%	37%	42%
Not helpful	0	16%	8%
Helpfulness in improving ability to prevent HIV			
Very helpful	82%	74%	78%
Somewhat helpful	12%	16%	14%
Not helpful	6%	11%	8%
Likelihood of recommending intervention to a friend			
Very likely	100%	84%	92%
Somewhat likely	0	16%	8%
Not likely	0	0	0
Applied to any of interventions’ texted job announcements			
Yes	24%	47%	36%
No	76%	53%	64%
Received any interventions’ texted jobs after applying^b^			
Yes	0	22%	15%
No	0	78%	85%
Preference for future number of text messages received			
Equal	71%	53%	61%
More	12%	21%	17%
Fewer	18%	26%	22%
Extent of liking weekly text message survey			
Liked a lot	35%	47%	42%
Somewhat liked	59%	47%	53%
Did not like	6%	5%	6%
Ease of responding to weekly text message survey			
Very easy	82%	84%	83%
Somewhat easy	12%	11%	11%
Not easy	6%	5%	6%

[a] Refers to job announcements only for comparison intervention and job announcement plus microenterprise activities for experimental intervention; [b] Denominator includes only participants who applied to one or more texted job announcements

### Acceptability of Educational Sessions & Mentoring

All experimental participants (100%, *n* = 17) who completed a post-intervention interview reported that they liked the microenterprise activities (e.g., educational sessions and mentoring) “a lot” compared to none (0%) who reported “somewhat liking” or “not liking” the microenterprise intervention (Table 2). In assessing the perceived effectiveness of the experimental intervention, 53% reported that the microenterprise activities were “very helpful” in improving their income-earning ability compared to 47% who reported that it was “somewhat helpful” and 0% who reported that it was “not helpful”. Eighty-two percent (82%) stated that the inclusion of HIV prevention education during sessions was also “very helpful” in improving their ability to prevent HIV compared to 12 and 6% who reported that it was “somewhat helpful” or “not helpful”, respectively. All experimental participants (100%) also stated that they were “very likely” to recommend EMERGE to a friend.

Table 3 describes qualitative likes and dislikes of the educational sessions and mentoring. Experimental participants most commonly liked discussing and asking questions on entrepreneurial topics, which they stated having had few prior opportunities to do (Table 3). Additional acceptable features were that the sessions were taught by friendly and patient facilitators who checked in on participants each week. Participants noted that the sessions helped them to “get on their feet” in becoming entrepreneurs and thinking about sensitive sexual and financial health goals in a safe environment.

“I was happy with the weekly check-in and telling what we’re spending [our] money on…getting a feel for each person and their businesses. And I liked asking questions on topics you don't really get to talk about…” – Woman, Experimental

“I liked that they [EMERGE facilitators] gave us tools and information to get us on our own feet but I think if you all do this again there should be more staff and more participants, like a bigger organization to help...” – Woman, Experimental

Dislikes of the educational sessions included interruptions by non-participants at the CBO site or interruptions by session peers who had poor engagement. A few participants also noted that determining which business to focus on was difficult to do, and they would have preferred more support from their mentors. Other dislikes were that EMERGE ended too soon, after 20 weeks, or that EMERGE met only once a week, rather than several times a week over a shorter period. Participants also suggested that a future program include more young adults and more guest speakers to provide more diversity and expand their peer and mentor networks. There was also interest in applying new skills from the sessions by mentoring future EMERGE participants, after they graduated.

“Education about starting a business was a good thing and I liked all the patience of the EMERGE team. Having sessions only once a week was okay… but it could have occurred more often than that to engage with participants more… basically meet more than once a week.” – Woman, Experimental

“The weekly groups based on entrepreneurship and guest speakers is what I liked the most but 20 weeks was really too long for the intervention... It should be group sessions about two times a week or something to make the intervention shorter…” – Woman, Experimental

“I couldn't figure out what business to start…that was hard. I needed more guidance.” – Male, Experimental

“I most dislike that it’s over… I wish graduates could continue with EMERGE as mentors or something for new members. It could be good for them and us.” – Man, Experimental

Mentors and CBO managers noted that they enjoyed being able to help participants pursue their business goals and felt the educational sessions and mentoring provided participants with something to look forward to. However, they also observed that some participants lacked the commitment needed to successfully manage a microbusiness, and, in some cases, did not feel they were able to provide sufficient support during and/or outside of the educational sessions.

**Table 3:** Participant, mentor, and manager responses to qualitative acceptability questions on intervention likes, dislikes, and recommendations in the EMERGE randomized feasibility trial

Topic	Codes	Rank	List of Findings	# stating
*Participants (N = 36)*				
Likes	Educational Sessions^a^	***	Getting a chance to talk about your business idea	8
		*	Receiving business information via handouts and presentations	2
		**	Talking to others with real microbusiness experience	4
		***	Friendly facilitators who created fun and hands-on environment	5
		**	Having opportunity to start a business / be an entrepreneur	4
	Grants & Incentives^a^	***	Valued having cash to help launch new business	7
		***	Obtaining additional money towards expenses from survey response	6
		**	Receiving a meal during sessions	4
	Informational Text Messages	***	Felt encouraged and inspired by weekly messages	14
		*	Repeated text messages on HIV reminded to get tested regularly	2
		*	Receiving weekly employment updates	2
	Text Message Surveys^b^	***	Asked important questions about sexual health and employment	7
		**	Text questions were inquisitive and straightforward	3
		*	Convenient timing of text surveys	1
	Misc^b^	*	Enjoyed talking to new people during interviews	2
		*	Rapid response of facilitators to questions or problems	1
Dislikes	Educational Sessions^a^	*	Interruptions by non-participants or unengaged participants	2
		*	Session times were difficult to match to personal schedule	1
		*	Business training too infrequent and too short to launch business	2
		*	Too few guest speakers and facilitators	1
	Grants & Incentives^a^	**	Business grants paid in installments were too small for large purchases	3
		*	Difficulty determining what to spend on microbusiness (how to start)	2
		**	Payments sometimes late and cumbersome to get	3
	Text Message Surveys^b^	***	Text surveys sometimes crashed or froze on cell phone	5
		*	Weekly text survey questions reminded of past difficulties	1
	Misc^c^	**	Disappointed to be assigned to less valuable comparison group	4
		*	Uncomfortable with teasing and complaints towards experimental participants	2
Recommendations	Educational Sessions^a^	*	Assign participants who complete EMERGE to be peer mentors	1
		*	Exclude less active participants and re-disperse their unused grants	2
		***	Offer more frequent sessions outside of work hours	5
		**	Include more mentors, facilitators, and participants during sessions	4
	Grants & Incentives^a^	**	Provide higher pay for responding to text survey	3
		*	Use a cash app or direct deposit to ease payment process	1
	Text Messages & Survey^b^	*	Provide more job announcements than one per week	1
		*	Ask different questions on different topics in text message survey	2
*Mentors and Managers (n = 7)*				
Likes	Recruitment	*	Perceived helpful interventions appealed to many young adults	2
	Implementation	**	Enjoyed being able to help participants pursue business goals	4
		*	Felt valued and well received by mentees/participants	1
		**	Saw that participants had incentives, something to look forward to	3
Dislikes	Recruitment	**	Some participants were mentally under-prepared at time of enrollment for business leadership, employment, or financial responsibilities	3
	Implementation	*	Unable to provide enough support to mentees given level of needs	2
Recommendations	Recruitment	**	Prior to enrollment, identify individuals with highest potential	3
		***	Facilitate more collaboration between mentors-mentees at enrollment	5
	Implementation	*	Provide updates to mentors about mentees when contact decreases	1
		*	Help apply to texted jobs or help to interview as a professional	1
		*	Support participants to purchase supplies via administrator or gift card	2

[a] Applicable to experimental participants only; [b] Applicable to all randomized participants; [c] Applicable to comparison participants only

### Acceptability of microbusiness Grants

Microbusiness grants were used by experimental participants to support their selected entrepreneurial activity during the intervention period. These activities included catering, apparel sales, cosmetic sales, entertainment and arts, and home deliveries. Having access to start-up resources was welcomed by nearly all experimental participants, particularly to purchase supplies relating to their microbusiness (Table 3). However, some participants reported that the amount of the grant was too small and the provision of partial installments hindered their ability to make significant purchases, such as for computing or digital devices. They also requested more rapid payment processing in the future. Very active participants also requested whether grant monies which were unused by less active or ineligible participants (e.g., due to not meeting the requisite milestone of a receipt or budget plan) could be re-allocated to their businesses in a future study trial. Participants also recommended having a project accountant provide more assistance to them in purchasing supplies and archiving receipts. Other recommendations included providing a larger microbusiness grant and using a cash or direct deposit, rather than paper-based checks to accelerate payment disbursements.

“It would’ve been good to have an accountant helping us participants…they could help with spending stuff and give guidance. And in my opinion there was not enough grant money dispersed you know… [It] could have been enough if [the] entire $1100 was given all at once…not little by little.” – Female, Experimental

“I suggest there be a way to put leftover grant money that was not given to inactive participants to [give to] active participants. That’s my advice.” – Female, Experimental

“…The opportunity to learn about how to start and run a business… broken down was really good for me. And I liked the food you all got every week for our sessions. …But in providing money for businesses, what I think is that EMERGE should have screened participants better.. for people who are serious about their business.” – Male, Experimental

### Acceptability of weekly text message survey

Forty-two percent (42%) of participants stated that they liked the weekly text message survey “a lot” compared to 53% who “somewhat liked” it and 6% who “did not like” it (Table 2). Most participants (83%) reported that the text message survey was “very easy” to respond to compared to 11% who said it was “somewhat easy” and 6% who said it was “not easy” to respond to. In addition, 74% of participants stated that responding to the text message survey was “very helpful” in improving their ability to prevent HIV compared to 16 and 11% who reported that it was “somewhat helpful” or “not helpful,” respectively. Although not an intended outcome of the survey, participants stated that the sexual and HIV behavioral questions reminded them to get tested regularly and to be safe. Other common “likes” for the weekly text message survey were that it was convenient, provided a small payment to responders, and reminded participants of their personal goals (Table 3). However, qualitative “dislikes” of the text message survey were the occurrence of technological challenges, such as crashing and freezing screens. Some participants also preferred greater payment for responding to the survey and use of different, rather than identical, questions each week.

“I liked that we got $3 weekly and being reminded by the questions to get tested regularly and all… But I think you all should implement the survey more than once a week…because it’s good to think about that stuff.” – Woman, Experimental

“I felt the surveys were important, and I liked the times when we received surveys. That was fine… And I liked the fast replies from EMERGE..” – Man, Comparison

“It was very inquisitive. It was very straight forward. The timing of the surveys (Friday mornings). But It wasn't diverse enough. More diversity. More in-depth questions (e.g., sexual preference questions, sexual orientation questions, etc.) – Woman, Comparison

“To be honest, I liked the survey questions specifically the HIV prevention stuff and that they catered to youth…like our long-term and short-term goals. But I think there could’ve been more questions... like better questions [or] …different types of questions instead of the same ones every week…” – Man, Comparison

“The survey payments were too little you know… I had to commute to pick them up and they were not even enough to cover transportation fares. So, yeah,… I think EMERGE should next time increase the survey payment amount.” – Woman, Comparison

### Acceptability of randomization

All enrolled participants (100%, *n* = 43) were willing to be randomized as part of the informed consent process (Fig. 1). However, 21% (n = 4) of comparison participants stated that they disliked the randomization process as it did not allow them to participate in their preferred experimental intervention, referred to by participants as “EMERGE-PLUS (+)” (Table 3). CBO managers also preferred that EMERGE be offered to all eligible participants rather than randomly assigning the more intensive microenterprise activities to half of the participants. Two experimental participants also indicated in the “other” category of dislikes that they did not like being teased by non-participants at the CBO for being assigned to the more rigorous experimental group. They also felt remorse from hearing complaints of non-selection by comparison participants. However, most participants accepted their randomization assignments given universal access to job announcements and survey payments regardless of group assignment. Our findings from both groups also indicated that while experimental participants shared microbusiness accomplishments to their colleagues and to peer comparison participants, specific EMERGE-PLUS(+) skills and materials were not shared.

“I did not get chosen for EMERGE+ because of the lottery selection…and that wasn’t good because it would have gave us an opportunity to become entrepreneurs.” – Woman, Comparison

“EMERGE did a good job incorporating sexual health and HIV info and stuff within surveys... But, I really think the EMERGE+ selection process should have been handled differently… Like, we did not like that people were randomly selected and all... In fact, a lot of people wanted to be a part of it and just didn't get the opportunity, you know? You should interview people just for EMERGE+…” – Woman, Comparison

“I really think you all should open the EMERGE+ program to more people, to allow them… to afford them the opportunity of business opportunity. That or just open up entirely… I don’t know how many you selected…but why not just open it up [to all]? …Some people who were really motivated did not get chosen.” – CBO Manager

### Process documentation of lessons learned

Table 4 summarizes process documentation findings relating to key successes, challenges, and potential modifications needed in a future effectiveness trial. Key successes were use of on-site recruitment and intervention implementation as well as racially-diverse, local business mentors and convenient text messaging assessments (Table 4). Participants appeared to value positive feedback during small-group setting to test products or give input on logos, business plans, names, and designs. Key implementation challenges related to varying levels of participation by participants during the latter half of the intervention, including disappointment by some randomization assignment. Changing employment schedules, low financial literacy, and cellular connectivity hindered session attendance and use of mentors and available business grants. Acceptability may be enhanced by assessing readiness at study enrollment, integrating more financial literacy training, and online educational options. Cluster randomization, peer referrals, or intervention wait-lists may also address dissatisfaction with randomization assignment and small sample size.

**Table 4:** Process documentation findings of intervention successes, challenges, and potential modifications of EMERGE feasibility randomized clinical trial

	Successes	Challenges	Potential modifications for effectiveness trial
**Recruitment**	On-site information and screening enhanced identification of participants.	Initial high motivation met by low engagement by some participants.	Assess readiness to start a business, such as referral or extended screening.
**Randomization**	Most participants completed steps for randomization eligibility.	Some participants were disappointed with randomization assignment.	Consider cluster randomization or wait-list control design.
**Intervention participation**			
Sessions	Sessions held at CBO at two different times with lunch and make-up materials.	Attendance declined over time with some training goals being too advanced.	More frequent, simplified sessions over shorter study period with travel supplement.
Grants	Prepared milestones and approved spending plan prior to disbursement.	Some funds were unspent due to low financial literacy in ordering supplies.	Provide more support for purchasing supplies and materials via accountant.
Mentors	Local and racially-diverse entrepreneurs matched to participants’ interests.	Schedules conflicted with sessions with limited ability to hire some participants, low trust.	Engage mentors at participant enrollment, using trust-building activities.
**Non-participation**	Minimized non-participation during first half of study (weeks 1 to 10).	Changing schedules or phone availability interrupted session attendance and survey completion, respectively.	Provide option to participate in asynchronous, online educational sessions and email/phone surveys.
**Text message survey**	Convenient and easy to use with weekly payment for response.	Malfunctioning phone and/or service at times with payment delays.	Include fewer questions over shorter study period with mobile payment.
**Sample size**	Appropriate for feasibility assessment and enabled personal attention.	Some participants wanted larger cohort to maximize peer interaction and group businesses.	Consider peer referral at study enrollment for larger effectiveness trial cohort.

## Discussion

Our results found that the study design and interventions was acceptable to the target population. Participants provided positive feedback regarding the experimental and comparison interventions and reported that they would recommend them to friends. We also observed other indicators of high acceptability. For example, comparison participants liked the job announcements and reported applying to and receiving jobs that were texted to them. Although not intended by the study team, comparison participants also described the weekly text message surveys as an active ingredient of the intervention that reminded them of financial and sexual health goals. We also found that experimental participants related to the information provided in the sessions and commented that they enjoyed learning about the personal experiences of local mentors and guest speakers. A few participants appeared to have high intrinsic motivation and requested more frequent sessions and a longer intervention period to further develop their businesses. Several experimental participants also reported wanting to receive equal number or more text messages (e.g., job announcements plus HIV prevention and business tips) in a future trial.

There are several potential factors which may have contributed to the high acceptability. First, high acceptability may be attributed in part to our development process, which included formative research regarding business training and design interests within the target population [19, 49], prior research regarding cell phone accessibility [50], asking participants during enrollment regarding the types of job announcements they would like to receive, and iteratively refining the educational sessions and text messaging content to include simple and applicable information.

A second potential explanation for the high acceptability is that the experimental intervention offered business and HIV prevention education with continued personalized feedback to participants during a vulnerable time period and included various tools for providing support such as handouts, sessions, text messages, mentors, and speakers. These tools were meant to assist participants in forming habits relating to healthy financial and sexual behaviors [51, 52]. The range of activities offered in the experimental intervention (e.g., educational sessions, mentoring, microbusiness grants, and job announcements) may also have led to overall acceptability. It is possible that acceptability for specific activities in the experimental intervention may have been directly or indirectly influenced by the specific activity’s being organized and integrated with other specific activities of the intervention. Therefore, the authors recommend that a future efficacy trial maintain the combination intervention model that was evaluated in this feasibility trial. For comparison participants, this continued feedback may have been experienced with the weekly text message communication on job openings during a period of un/under-employment.

Third, participants may have responded positively because such an intervention is uncommon among racial minority communities for young adults. Therefore, participants may have placed more value on the interventions in a pilot test setting. In addition, during enrollment, we described both interventions as novel activities aiming to improve employment for young adults, which may have enhanced participant acceptability of randomization to experimental or comparison intervention. Taken together, these positive assessments suggest microenterprise and other microeconomic interventions are acceptable ways of providing vulnerable young adults with important financial and sexual health content, in a manner that addresses HIV risks associated with economic vulnerability. Participants appeared to be motivated to engage with the interventions because of their economic empowerment potential and valued the integration of HIV prevention education.

However, the study identified some barriers that may have hindered acceptability. First, participants reported mixed acceptability for the weekly text message survey due to usability issues from bugs in the survey that did not occur during the beta-testing of the text messaging platform [53]. More research is needed to understand potential compatibility issues with various cellular or text messaging systems. Some participants were also dissatisfied with perceived low and delayed payments for completion (e.g., two to five days later) or use of the same survey questions each week. We initially used a weekly text message survey to more frequently and more conveniently assess study outcomes than was possible with conventional pre-post designs. However, decreasing the duration of the assessment period and the number of weekly questions may enhance acceptability. Immediate payment via a mobile payment service may also provide a more meaningful reward. Secondly, there was mixed acceptability regarding the role of mentors. Some mentors recommended greater support to foster mentor-mentee relationships, such as meeting participants when they enrolled in the study rather than a few weeks later and having more collaborative tasks to pursue together. Assessing readiness to start a microbusiness and integrating more employment readiness training into the study’s educational sessions was also seen by other mentors as a way of enabling more productive relationships with participants. In addition, although rare, being teased or hearing complaints by peers who were not in the experimental group was a barrier to acceptability for some participants. Decreasing the intervention’s publicity during recruitment, offering a network-based intervention to minimize contact between study arms, including wait-list option, or providing guidance to all CBO youth regarding interactions with study participants may be an important addition.

Finally, it is worth discussing this study’s secondary acceptability findings as compared to the level of participation reported in our previously published primary outcomes manuscript, in which the study initially observed moderate to high participation that declined among some participants over time [38]. The complex relationship of acceptability and participation has been documented in prior studies and suggests that acceptability is impacted by multiple factors, including individual circumstance and changes over time [54]. In this study, high acceptability may have reflected participants’ flexibility to engage in the intervention at their discretion and participate in response to their changing preferences and situations. We found that low participation was not associated with feelings of non-acceptability, but more commonly attributed to external factors such as personal issues relating to scheduling, bereavement, or housing conflicts and instability. However, it is also possible that participants with low engagement had decreasing acceptability in considering the intervention commitment too long or demanding. Reducing commitment expectations may be an important determinant of acceptability for some participants. Enabling participants to recruit eligible peers to join them in the intervention may also enhance acceptability, particularly for young adults who may benefit from additional peer support when experiencing external challenges [55].

### Limitations and strengths

The limitations of this study are worth noting. We do not know acceptability for two randomized participants who were lost to follow-up. While reasons for loss to follow-up may reflect factors external to study’s methods [54], it is also possible that low acceptability could have moderated retention in the study. In addition, participants may also have been reluctant to express negative views regarding an intervention perceived as a unique economic opportunity. Finally, although the study obtained feedback on the acceptability of a range of intervention activities, such as educational sessions or job announcements, acceptability of more detailed intervention components, such as a specific text message or activity within an educational session, were not assessed. Future studies may involve repeated and in-depth acceptability measures. Important strengths of the study include its inclusion of participants and stakeholders in understanding acceptability and documentation of perceived implementation barriers and facilitators by the study team. Additional study strengths included use of qualitative and quantitative measures and recommendations for conduct of future similar trials.

## Conclusion

Microeconomic interventions are acceptable ways of providing young adults with important financial and sexual health content to address HIV risks associated with economic vulnerability. Our findings indicate acceptability of the interventions and outcome assessments, providing important guidance for the development of a future trial to test effectiveness. The largely positive endorsement suggested that factors contributing to acceptability included perceived economic potential, sexual health education, convenience, incentives, and personalized encouraging feedback to participants. Improving text messaging functionality and online and in-person intervention support may enhance acceptability. Use of peer referral, wait-list, or network designs may also improve participant acceptability and overall experience in a definitive trial.

## Supplementary Information

**Additional file 1.** Acceptability Questionnaire.

## Data Availability

The data that support the findings of this study are qualitative transcripts and notes and are therefore not publicly available due to their containing information that could compromise participant privacy.

## Abbreviations

AIRS: AIDS Interfaith Residential Services
EMERGE: *E*ngaging *M*icroenterpris*E* for *R*esource *G*eneration and Health *E*mpowerment
CBO: Community Based Organization
HIV: Human Immunodeficiency Virus
IRB: Institutional Review Board
MD: Maryland
USD: United States Dollar
YO!B: Youth Opportunities! Baltimore
