# Supplementary material for: Acceptability of a feasibility randomized clinical trial of a microenterprise intervention to reduce sexual risk behaviors and increase employment and HIV preventive practices (EMERGE) in young adults: a mixed methods assessment
Source: BMC Public Health. 2020 Dec 2;20:1846. doi: 10.1186/s12889-020-09904-x (PMC7709242; doi:10.1186/s12889-020-09904-x)
Supplement: Supplementary file 1 — Additional file 1. Acceptability Questionnaire. [file 12889_2020_9904_MOESM1_ESM.docx]

**IN-PERSON SURVEY**

The EMERGE PROJECT

**Study Title: Feasibility Trial to Reduce Sexual Risk Behaviors in African-American Young Adults,**

**Aged 18 to 24, Who Are Out of School, Unemployed, and**

**Experiencing Homelessness in Baltimore City, Maryland, United States**

Endline Only Process Measures – Qualitative and Quantitative

**Part 1: Open-Ended Questions**

1. What did you like most about being a part of the EMERGE Project?
2. What did you dislike most about being a part of the EMERGE Project?
3. If you had to be in EMERGE again, what would you change about the program?

**Part 2: Close-Ended Questions**

| Q# | Question | Coded Response |
| --- | --- | --- |
| 1. | Have you been receiving the EMERGE weekly text message surveys? | 01 Yes 02 No 88 DK/NR |
| 2. | When you receive a survey, do you usually respond? | 01 Yes 02 No 88 DK/NR |
| 3. | What are some of the reasons why you have not been able to respond or why you might decide to skip a survey? | 01 Didn’t want to  02 Forgot to  03 Didn’t have phone  04 Problem with phone  05 Survey fatigue  06 Privacy concerns  07 Other _______________  88 DK/NR 99 N/A |
| 4. | Have you been receiving the weekly text job announcements? | 01 Yes 02 No 88 DK/NR |
| 5. | Have you applied to any of the job announcements that were texted to you? | 01 Yes 02 No 88 DK/NR |
| 6. | Have you received any of the jobs that you applied for that were texted to you? | 01 Yes 02 No 88 DK/NR |
| 7. | Have you been receiving the EMERGE HIV prevention text messages? (i.e., weekly tips) | 01 Yes 02 No 88 DK/NR |
| 8. | Has your cell phone number changed since the beginning of the EMERGE project? | 01 Yes 02 No 88 DK/NR |
| 9. | Are you still using the same cell phone number registered to this project? | 01 Yes 02 No 88 DK/NR |
| 10. | What are the reasons why your cell phone number has changed? [Select all that apply] | 01 Lost phone  02 Stolen phone  03 Switched cellular provider  04 Avoiding phone harassment  05 Subscription expired  06 Stopped payments  07 Got a new phone  08 Other ____________________  88 DK/NR 99 N/A |
| 11. | Do you have more than one cell phone that you regularly use? | 01 Yes 02 No 88 DK/NR |
| 12. | Do you have unlimited text messaging? | 01 Yes 02 No 88 DK/NR |
| 13. | Has any of your friends or family read some of the text messages you have received from EMERGE? (e.g., includes survey, job announcements or HIV messages) | 01 Yes 02 No 88 DK/NR |
| 14. | Have you had any positive experiences as a result of receiving an EMERGE text message? | 01 Yes 02 No 88 DK/NR |
| 15. | What kind of positive experiences have you encountered? [Select all that apply] | 01 Earned money/job  02 Received encouragement  03 Talked with sexual partner  04 Talked with friend  05 Talked with counselor/mentor  06 Acquired HIV products  07 Acquired HIV test  08 Made a new friend  09 Felt happy/inspired  10 Other ________________  88 DK/NR 99 N/A |
| 16. | Have you had any negative experiences as a result of receiving an EMERGE text message? | 01 Yes 02 No 88 DK/NR |
| 17. | What kind of negative experiences have you encountered? [Select all that apply] | 01 Lost access to phone  02 Criticized or teased  03 Responded to suspicion  04 Experienced violence  05 Felt sad or discouraged  06 Dispute or conflict  07 Other ________________  88 DK/NR 99 N/A |
| 18. | Have you had any difficulties reading or understanding the EMERGE text messages? | 01 Yes 02 No 88 DK/NR |
| 19. | Would you like to receive the same, more, or less text messages from EMERGE? | 01 Same  02 More  03 Less  88 DK/NR 99 N/A |
| 20. | About how much do you spend each month on cell phone costs? | ____________ USD $ dollars/month |
| 21. | How much did you like the intervention? | 01 Liked a lot  02 Liked a little  03 Did not like at all  88 DK/NR 99 N/A |
| 22. | How helpful to you was the intervention in improving your ability to earn income? | 01 Very helpful  02 Somewhat helpful  03 Not helpful at all  88 DK/NR 99 N/A |
| 23. | How helpful to you was the intervention in improving your ability to prevent HIV? | 01 Very helpful  02 Somewhat helpful  03 Not helpful at all  88 DK/NR 99 N/A |
| 24. | How likely are you to recommend the intervention to a friend? | 01 Very likely  02 Somewhat likely  03 Not likely at all  88 DK/NR 99 N/A |
| 25. | How much did you like the text message surveys? | 01 Liked a lot  02 Liked a little  03 Did not like at all  88 DK/NR 99 N/A |
| 26. | How easy was it to respond each week to the text message survey? | 01 Very easy  02 Somewhat easy  03 Not easy at all  88 DK/NR 99 N/A |
